# Four Principles of Transformative Adaptation to Climate Change-Exacerbated Hazards in Informal Settlements

**DOI:** 10.1002/wcc.70008

**Published:** 2025-05-05

**Authors:** Ben C. Howard, Simon Moulds, Samuel Agyei-Mensah, Khadiza Tul Kobra Nahin, Zahidul Quayyum, Brian E. Robinson, Wouter Buytaert

**Affiliations:** 1 Department of Civil and Environmental Engineering, Imperial College London, London, UK; 2 School of GeoSciences, University of Edinburgh, Edinburgh, UK; 3 Department of Geography and Resource Development, University of Ghana, Accra, Ghana; 4 James P Grant School of Public Health, BRAC University, Dhaka, Bangladesh; 5 Department of Geography, McGill University, Montreal, Canada

**Keywords:** climate change adaptation, disaster risk reduction, informal settlements, sustainable development, transformative adaptation

## Abstract

Residents of urban informal settlements are among the most at-risk of climate change-exacerbated hazards. Yet, traditional approaches to adaptation have failed to reduce risk sustainably and equitably. In contrast, transformative adaptation recognizes the inextricable nature of complex climate risk and social inequality, embedding principles of social justice in pathways to societal resilience. Its potential for impact may be greatest in informal settlements, but its application in this context introduces a new set of challenges and remains largely aspirational. To address this missed opportunity, in this focus article we provide clarity on how transformative adaptation can manifest in informal settlements. Although context-dependency precludes the formulation of specific guidelines, we identify four principles which are foundational to its deployment in these settings. Acknowledging constraints, we define levels of achievement of the principles and suggest how they might be reached in practice. Achieving transformative adaptation in informal settlements is complex, but we argue that it is already achievable and could represent a prime opportunity to accelerate the rate of adaptation to build a climate resilient society.

## 1 Introduction

Climate change disproportionately impacts those living in poverty, exacerbating profound social inequalities (Islam and Winkel 2017). Some of the most underprivileged people live in informal settlements, which here we define as residential areas that develop outside of formal planning and regulation in towns and cities and are characterized by tenure insecurity, socio-economic marginalization, lack of infrastructure and services, and unsafety including high exposure to environmental and health hazards (Satterthwaite et al. 2020). More than 1 billion people currently live informally, with this figure expected to rise as people continue to migrate to cities (Satterthwaite et al. 2014). Around 90% of this projected growth will occur in Africa and Asia, which are also disproportionately exposed to the effects of climate change (UN Population Division 2018).

Residents of informal settlements are particularly at risk of weather and climate extremes, which are expected to become more severe in our changing climate (Satterthwaite et al. 2020). Not only are informal settlements concentrated in hotspots of extremes, but their residents are also highly vulnerable to negative impacts. Vulnerability is driven by crowded and poorly constructed housing, insecure tenure of housing often in hazardous areas, inadequate hazard protection infrastructure, limited access to water and sanitation (Kashem et al. 2022), a lack of access to a variety of social services, and a lack of democratic voice (Herslund et al. 2016). These experiences result in large in-equalities in the levels of climate risk experienced by formal and informal communities, even those that are similarly exposed to hazards, and increase the impact on society (Lindersson et al. 2023).

**Figure 1:**
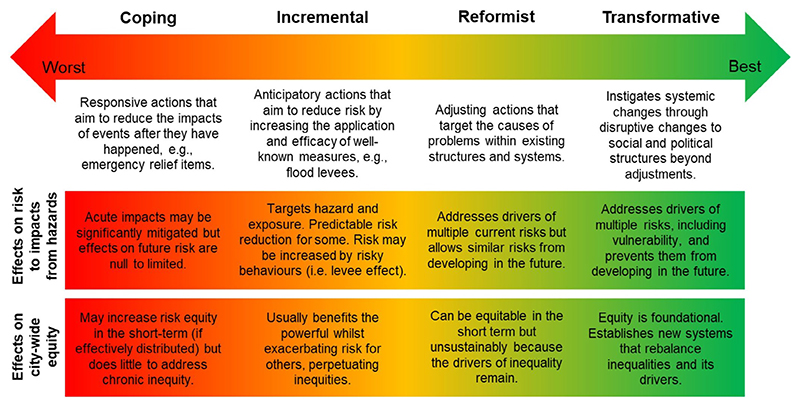
Adaptation strategies and their effects on climate risk and equity. Coping, incremental, reformist, and transformative approaches are conceptualized on a continuum from limited climate risk reduction or increases to equity to transformative change toward equitable climate resilience.

Despite the elevated risks experienced by residents of informal settlements, they have typically benefitted least from climate adaptation programs and policies (Dottori et al. 2018). Current approaches to climate adaptation generally fall into three categories: coping, incremental, and reformist (Figure 1). Coping strategies are reactionary, aiming to reduce the impacts of events that have already happened; incremental strategies are anticipatory, aiming to build resilience to future climate events by making adjustments using established measures (Liu et al. 2018); reformist strategies aim to target the cause of the problem but without addressing the systems that enable and up-hold them (Heikkinen et al. 2019; Singh et al. 2024). To varying degrees, these approaches are criticized for failing to tackle the systemic causes of vulnerability, serving to maintain the status quo (Singh et al. 2024), promoting maladaptation that perpetuates existing social inequalities (Reckien et al. 2023), and missing opportunities to realize substantive benefits for human development (Williams et al. 2019). For example, the financial damages suffered by the urban poor as a result of climate extremes usually represent a larger proportion of their economic capital compared to wealthy people, yet they often receive the least financial assistance to recover (Dottori et al. 2018). There is an urgent need to enable better strategies that provide equitable, sustainable, and holistic adaptation which centers on the most vulnerable.

Transformative adaptation is an emerging strategy that recognizes the inextricable linkages between climate vulnerability and social inequality (Anguelovski et al. 2016). It seeks to understand the systemic causes of vulnerability and how these manifest in an accumulation of risk for different groups (Few et al. 2017). It aims to address these root causes by going beyond making adjustments to existing systems to meet climate change conditions, especially using structural measures (e.g., infrastructure upgrading) that have typically characterized incremental adaptation and slum upgrading (Kates et al. 2012), by instead instigating systemic societal change (Heikkinen et al. 2019). Deployed effectively, it can initiate a shift in the development trajectory of a community toward a more equitable, sustainable, and climate resilient future (Fedele et al. 2019).

The potential of transformative adaptation to increase societal resilience to climate change may be greatest when applied in informal settlements in the global South (Nagendra et al. 2018), because they are hotspots of climate risk (De Risi et al. 2013), disbenefit from other adaptation strategies (Ziervogel 2021), and represent the bottom of the socioeconomic spectrum, driving higher inequality which increases societal risk (Lindersson et al. 2023). However, in doing so, a different set of challenges may be introduced because informal settlements are typically excluded from the frameworks used by institutions and governments to organize and affect change; they are subject to multiple and more severe threats to wellbeing, and the relationship between informal residents and governments is often complex and difficult. For this reason, despite the large potential benefits, transformative adaptation in informal settlements remains largely aspirational.

**Figure 2:**
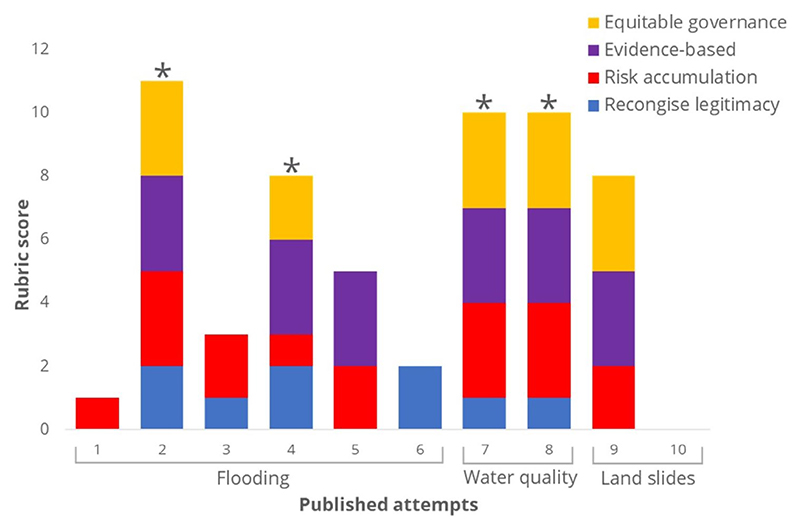
Outcomes of applying the four principles rubric (Figure 3) to published attempts of transformative adaptation in informal settlements. * = Successful attempts (i.e., transformative). Numbers on the *x* axis pertain to case-studies, 1 (Cobbinah et al. 2022), 2 (Fox et al. 2021), 3 (Mitra et al. 2017), 4 (Linh and Quan 2020), 5 (Linh and Quan 2020), 6 (Simarmata and Surtiari 2020), 7 (Brown 2018; French et al. 2021; Revitalising Informal Settelements and Their Environments 2021), 8 (Leder et al. 2021; Reidpath et al. 2022), 9 (Hostettler et al. 2019), 10 (Anguelovski et al. 2016).

The aim of this focus article is to encourage and inform attempts at transformative adaptation to climate-exacerbated hazards (floods, droughts, landslides, heatwaves, and water pollution events) in informal settlements by outlining the primary challenges to its implementation and providing actionable routes to address them. We focus on adaptation to hazards rather than to broader climate risk. It is not our aim to provide an exhaustive literature review. Instead, we have sought to identify the most relevant literature, particularly focusing on documented case studies of attempts at transformative adaptation in informal settlements. A paucity of examples was revealed: only 10 case studies in total and none for drought or heatwaves (Figure 2). For example, a Web of Science search (07/02/2025) using search terms “informal settlement*” AND “transformative adaptation” OR “transformational adaptation” (All Fields) returned only five examples. A wider search using search terms “informal settlement*” AND “climate adaptation” OR “climate change adaptation” (All Fields) returned more results (92), but most of which pertained to coping or incremental adaptation—not directly to transformative adaptation which was the focus of this paper. Although we found case studies elsewhere, we also draw from the broader climate change adaptation and urban development literature, at the same time avoiding replicating the several comprehensive advanced reviews and overviews that have been published on these broader subjects (e.g., Tenzing 2020; Woroniecki et al. 2019; Nightingale et al. 2022; Newell et al. 2021; Ajulo et al. 2020).

Providing prescriptive instructions on transformative adaptation in informal settlements is precluded by inherent context dependancy (Dottori et al. 2018), which demands case-by-case design of policies and interventions. However, by discerning some common features of successful climate adaptation programs across a range of development scenarios, we aim to provide a starting point for academics, policy makers, and practitioners to develop and deploy transformative approaches in these settings. Reviewing the available case studies and adjacent literature, we identified four principles which we find to be foundational in enabling transformative adaptation to climate change in informal settlements. These are: improve tenure security; ensure governance frameworks are equitable and fit for purpose; generate and use data for evidence-based decision making; and address the drivers of complex risk. In every documented case study where transformative adaptation was achieved, these principles were achieved at least to a minimum degree (Figure 2) (methodological detail in the Supporting Information). We organize our focus article around these four principles to make our contribution actionable—that is, directly usable to inform decisions.

## 2 Improve Tenure Security

A defining characteristic of informal settlements is the occupation of land to which the residents have no legal claim (Satterthwaite et al. 2020), leading to a substantial risk of being unwilfully displaced from their homes (i.e., land tenure insecurity) (Baumgartner et al. 2022). Tenure insecurity has well-established negative implications for the physical and mental health of residents and serves to repress livelihoods and poverty reduction (Baumgartner et al. 2022), amplifying risk by increasing the vulnerability of residents to climate hazards. It also contributes to the perception that informal settlements are temporary, discouraging investment and preventing long-term commitments (Fox et al. 2021). For example, in Bangladesh, the lack of relevant legislative frameworks limits government support to residents of the informal settlements (Hossain and Rahman 2017). Insecure land tenure can also lead to eviction and resettlement, isolating individuals from economic opportunities and important social structures while typically doing little to curb informal development at the city level (Anguelovski et al. 2016; Few et al. 2017; Nikuze et al. 2022). Alternatively, governments may simply ignore informal communities, a dichotomy that is seen in Accra, Ghana, where the government approach has been characterized as varying between “convenient absence” and “aggressive presence” (Amoako 2016). The lack of tenure rights is often linked with broader issues of citizenship, which may particularly affect migrant populations who lack formal recognition as city residents (Michael et al. 2018; Singh and Basu 2020).

Efforts toward transformative adaptation must aim to improve tenure security for residents of informal settlements (Zazyki et al. 2022). One option is to grant informal households legal rights to land ownership (i.e., titling). This can increase investment in housing and services (Awunyo-Akaba et al. 2016), facilitate social mobility, and help avoid overcrowding (Galiani and Schargrodsky 2010). In Brazil, the 1988 Federal Constitution established as a general principle the right to regularize many informal settlements, resulting in greater tenure security for many informal residents and widening access to vital public services, thereby increasing their resilience to climate hazards (Fernandes 2007). However, while this approach may sometimes be optimal for residents, it is rarely feasible over the short to medium term, even for legitimate and motivated governments, and can also pose risks to both parties (Zazyki et al. 2022). For residents, formalizing ownership can increase maintenance, administration, rent, and servicing costs (Linh and Quan 2020), and lead to gentrification and, ultimately, poor households being priced out of the area (i.e., market eviction) (Baumgartner et al. 2022). For governments, on the other hand, granting legal rights to ownership can imply a duty to provide services and infrastructure that, in effect, promotes the continued occupation of risky areas and can generate inflationary pressures and economic volatility (Durand-Lasserve et al. 2002). On its own, titling may not enhance tenure security, which is also closely tied to improved access to social benefits and better environmental conditions (Almansi 2009). Residents experience these factors in diverse ways, depending on intersecting social, economic, and political circumstances (Singh and Basu 2020), highlighting the need to recognize the heterogeneity of informal communities when assessing tenure security and its broader impacts.

When the risks of titling or regularization are high, improved land tenure security can be realized by other means. Collective ownership (e.g., community land trusts) may secure housing tenure by allowing members to hold long-term leases and, unlike many other affordable housing options, allow wealth generation and facilitate social cohesion (Hindman and Pollack 2020). A community land trust was established in an informal settlement in San Juan, Puerto Rico, and, along with flood mitigation measures, resulted in a safer and more desirable community, as well as a higher land value that benefited residents (Davis and Fernández 2020). Even though collective ownership may not incentivize investment to the same extent as traditional individual ownership, and management systems must overcome free-rider issues, positive examples of collective governance are common (Baumgartner et al. 2022). Another mechanism to improve tenure security is via usufructuary rights, which can provide temporary rights for current residents to inhabit and/or derive income from land without legal ownership while preventing, for example, the sale or transfer of rights to another (Galiani and Schargrodsky 2010). In some instances, these rights can provide enough tenure security to foster investments and developments (Fenske 2011), but they can also repress wealth accumulation and transfer for poor households and lead to overcrowding (Galiani and Schargrodsky 2010). Merely increasing residents’ perception of tenure security can have profound effects on their health and wellbeing, and the development trajectories of informal settlements (Baumgartner et al. 2022). Perceived tenure security is subjective and household-specific, but related to local political, economic, and sociocultural contexts (Baumgartner et al. 2022), and in general can be improved by upgrades to housing, services, and environmental conditions (Almansi 2009), enhanced neighborhood safety, and better access to local economic and educational opportunities, whether they are driven by residents or organized externally (Robinson et al. 2018). Moreover, educating residents on their rights to inhabit land and demand services can empower them to assert their claims, thereby increasing tenure security in practice (Awunyo-Akaba et al. 2016).

Other mechanisms to increase tenure security in informal settlements include engaging residents in local governance, which can empower individuals to demand better services and infrastructures, reducing exposure to climate (e.g., flooding and heatwaves) and non-climate (e.g., crime and violence) risks (Baumgartner et al. 2022). This engagement can additionally form a basis for developing trust between formal and informal sectors, and the possibility for informal communities to benefit from progressive risk reduction policies. Trusting relationships may also be instigated through government investment, which can signify a commitment to risk reduction and improve perceptions of tenure security. For example, in Rosario, Argentina, where formal land-titling was unrealistic within existing legal frameworks, government-supported upgrading programs in themselves substantially reduced the perceived threat of eviction, thereby contributing to greater tenure security in practice (Almansi 2009). Resettlement represents a significant challenge but may sometimes be necessary (Filho et al. 2023), for example, where sites of informal settlements are severally exposed to climate hazards (Kates et al. 2012). In this instance, transformative approaches must seek to understand the root causes of informal occupation of the high-risk area. Residents of informal settlements are often subject to multiple acute risks, including inadequate access to shelter, food, drinking water, and energy, and may be willing to accept a higher yet seemingly more distant risk of climate hazards to meet their immediate needs (Satterthwaite et al. 2020). Alternative accommodation must address acute and competing risks, for example, by providing suitable transport options to access economic opportunities. Resettlement is additionally undesirable because it will usually exacerbate the challenge of providing suitable and affordable housing that afflicts many towns and cities globally, and of which it is already a symptom (Anguelovski et al. 2016).

## 3 Ensure Governance Frameworks Are Equitable and Fit for Purpose

Transformative adaptation requires meaningful contributions from a range of formal and informal actors who must jointly develop courses of action that equitably serve the interests of residents of informal settlements and align with the priorities of city, national, and international governments (Pelling and Garschagen 2019). Placing stakeholders at the center of decision-making ensures that outcomes are salient and acceptable, leverages local knowledge and experiences, and empowers local, small-scale adaptation (Ziervogel 2021; Susskind and Kim 2022; Petzold et al. 2023). However, by themselves, bottom-up approaches often pertain to incremental or coping strategies because they are usually conducted on small scales (e.g., household or neighborhood) and fail to address the drivers of risk (Cobbinah et al. 2022). Cross-scale integration to link grass-roots adaptation efforts with regional and national adaptation plans and/or climate policymaking is needed to ensure equitable governance and maximize the impact of bottom-up adaptation strategies in informal settlements (Williams et al. 2019; Fünfgeld 2010). Capable and empowered local government is usually a critical partner in driving transformative adaptation, hosting knowledgeable and experienced individuals who are situated at the closest interface to citizens (Susskind and Kim 2022; Williams et al. 2018). By bridging the gap between local initiatives and higher levels of governance, local governments can ensure that resources, capacities, and funding are appropriately allocated to support adaptation efforts at the grassroots level. Furthermore, leveraging resources and expertise from private and third sectors may accelerate adaptation and stimulate local economic development (Rojas 2019; Satterthwaite 2017).

Establishing equitable governance frameworks that support contributions of necessary actors is vital but often remains aspirational (Petzold et al. 2023). At the institutional level, branches and levels of government often work in isolation, which is counterproductive for dealing with the combined impacts of climate change and social development, which transcend local scales and the briefs of individual government ministries (Satterthwaite et al. 2020; Williams et al. 2018). Creating an environment in which state and non-state (e.g., community groups and non-governmental organizations) can effectively and equitably work together with residents also represents a challenge, especially considering that informal settlements themselves can be highly heterogeneous (Deshpande et al. 2018). Selecting appropriate formats and frameworks in which to arrange climate adaptation can help to facilitate these complex interactions. Institutional arrangements, governance frameworks, and collaborative processes, including the organizational structures of teams, modes of communication and decision making, and units of spatial planning, should be tailored to existing partner dynamics and local preferences. Failing to do so risks disrupting existing support and governance systems, which can contribute significantly to climate risk mitigation (Mitra et al. 2017). For example, in one project in Kibera, Kenya, governance frameworks were forced (i.e., insensitively translated) from community-relevant formats to state-relevant formats (e.g., enumeration areas) which disrupted naturally homogeneous groups and instead defined groups by socially irrelevant geographical parameters (Mitra et al. 2017).

By contrast, successful attempts at facilitating transformative adaptation include early, meaningful, and continual consultation with residents. On another project in Kibera, consultation with residents ensured the success of a flood adaptation project that reduced risk equitably, and also reduced crime and conflict (Mitra et al. 2017). Similarly, participatory budgeting in Porto Alegre, Brazil allowed informal communities to work with local governments to define their needs and priorities and manage the implementation of public works (Cabannes 2015). They also develop mechanisms for social accountability between actors, including democratic accountability of local and city governments. In Kenya (Mitra et al. 2017) and Brazil (Fernandes 2007) these accountability mechanisms were important for building trust and strengthening partnerships. Such policies demonstrate commitment to co-production and instigate trust, enabling the co-development of inclusive and efficient policies which reduce risk and create opportunities for transformative adaptation (Pelling and Garschagen 2019).

While residents can be motivated to engage in adaptation activities, for example driving bottom-up strategies, formal actors must be intentional about including a range of citizens. Relationships between authorities and citizens should extend directly to as many inhabitants of informal settlements as possible to capture the significant heterogeneity that often exists within them (Michael et al. 2018). This heterogeneity can result in stakeholders experiencing very different outcomes from the same interventions, even at the household level, as vulnerabilities are shaped by various intersectional factors including gender, class, and race (Singh and Basu 2020). Consequently, representative inclusion (e.g., by a community leader) should be minimized because it can perpetuate inequalities within the settlement (Simarmata and Surtiari 2020). Furthermore, mistrust and resentment of formal actors by some residents is common, for example resulting from historic malpractice by local authorities, such as forced eviction and the failure to respond to disasters (Mitra et al. 2017). These dynamics present a challenging basis on which to begin productive discussions. Relationship building in these contexts may be catalyzed by specialists: in Ho Chi Minh City, Vietnam, social workers were successfully employed to help bridge existing gaps between authorities and communities (Linh and Quan 2020). Relationship and trust building take time in any instance, and funders should allow project durations that afford time for these activities.

## 4 Generate and Use Data for Evidence-Based Decision Making

Evidence-based decision making is essential to design interventions that are effective at minimizing risk now and in the future, equitably serve the interests of multiple stakeholders, and limit unforeseen consequences and externalities (Dodman et al. 2019). Without evidence, it is not possible to reliably predict the effects of risk reduction interventions or to adopt adaptive approaches that respond to inefficiencies, new opportunities, or non-stationarities (e.g., in climate or societies). Evidence can be generated using many types of information, including quantitative, qualitative, and experiential. However, there is a severe paucity of data and information on and about informal settlements, seriously hindering evidence and knowledge generation and precluding adaptive and responsive management (Osuteye et al. 2017; Chakraborty et al. 2015; Hofmann et al. 2015). The improvised nature of informal settlements and attitudes of decision-makers toward them (e.g., ignorance or avoidance (Amoako 2016)), can lead to their exclusion from data collection exercises (e.g., censuses), resulting in a lack of documentation of their existence, let alone specific needs and vulnerabilities, and rendering them invisible to authorities (Satterthwaite et al. 2020). New innovations and technologies are transforming data acquisition and knowledge generation and opening opportunities to facilitate evidence-based decision making in informal settlements. For example, open source data (e.g., satellite imagery, street view imagery), coupled with artificial intelligence processing and analyses techniques, is enabling the mapping and characterization of informal settlements around the world, for example in the Middle East (Fallatah et al. 2019), South East Asia (Assarkhaniki et al. 2021), East Africa (Mahabir et al. 2020), and India (Kohli et al. 2016).

Monitoring and forecasting environmental hazards remains a challenge, in particular at the local scale, but opportunities to reduce the data gap are rapidly emerging. New technologies are improving the accessibility of monitoring and modeling of hazards. For example, low-cost and open-source solutions enable non-specialists to deploy sophisticated monitoring networks for a fraction of the cost of commercial alternatives, which can provide data on hazards, especially when combined with real-time data transmission (e.g., using internet of things (IoT) technologies (Mei et al. 2020)). Among other variables, these technologies can collect high-resolution and real-time data on precipitation and river water levels, which can be used to model flood hazard and exposure down to the neighborhood level. They can also be used to inform flood early warning systems, like in Honduras, where such systems were co-designed with communities in nine informal settlements, coupled with community engagement, education, and planning activities (Peters et al. 2022). IoT networks have also been used to inform landslide early warning systems, for example in a mountainous area in Medellin, Colombia, which used a network of low-cost geosensors to collect data on groundwater and minute subsurface movements (Gamperl et al. 2021). The open-source nature of these technologies provides unprecedented flexibility, allowing solutions to be tailored to the needs and preferences of communities and opening opportunities for citizens to participate in innovation (Buytaert et al. 2016). Moreover, other forms of participatory data collection (e.g., citizen science) can enable high spatial and temporal resolution data collection of community-relevant parameters (Buytaert et al. 2016). These efforts can allow novel forms of governance to emerge by empowering communities to join in evidence-based decisions on climate adaptation (Buytaert et al. 2016) and provide the basis for collaboration between communities and the government on planning and adaptation (Patel et al. 2012; Wolff et al. 2021).

Demographic data is necessary to understand the heterogeneity of vulnerability within informal settlements, which is driven by exposure to hazards and susceptibility to negative effects, but mitigated by a capacity to resist or respond (Satterthwaite et al. 2020). Detailed information on these fundamental drivers is necessary for interventions to target relevant root causes of risk for individuals and to ensure equity in outcomes. Such data are usually collected in national censuses, but informal settlements are often neglected from the process of designing and implementing these surveys, leaving them excluded from important data that is widely used to make decisions (Satterthwaite et al. 2020). Moreover, as these surveys are typically conducted on multiyear cycles, the resulting data can soon become unreliable in dynamic communities like many informal settlements. Alternatively, targeted surveys and questionnaires facilitated by the communities themselves can provide valuable socioeconomic data. For example, the community-based NGO Shack/ Slum Dwellers International has worked with governments to conduct enumerations and gather household-level demographic data (Patel et al. 2012). NGOs are also helping to close the gap on demographic data availability in informal settlements, such as Prindex, which conducts surveys on land and housing security globally and provides the data for free and open access (Prindex, n.d.). Complementing this type of manual data collection, novel methods using open-source imaging and machine learning techniques are unlocking a new era in high-resolution social data that could prove transformative in generating evidence bases for adaptation (Nathvani et al. 2023).

Increasing data availability could improve the deployment of multiple adaptation strategies and is critical in enabling transformative adaptation. A primary coping strategy is the distribution of emergency relief items, such as food, water, and medicines, which could be sped up and made more equitable by basing rapid needs assessments on real-time hazard data. Improved data availability may also widen the availability of financial products to residents of informal settlements, who have typically been excluded without a legal address. For example, it could enable the provision of index insurance, an innovative approach that pays out based on a predetermined index that is related to loss and damage. The payout mechanism is better suited to informal settlements because it substantially reduces the transaction costs associated with traditional insurance (Linnerooth-Bayer et al. 2011). Having access to financial services such as index-based insurance could help victims of climate shocks recover quickly and provide security to investment, avoiding entrapment in a cycle of shock-recovery and empowering people to break free from poverty (Fedele et al. 2019).

Data collection and sharing should be part of a continuous, long-term relationship between communities and local governments. Long-term monitoring arranged without the participation of communities could be perceived to violate privacy. Furthermore, while data sharing are essential for equitable and informed decision making, it must be organized sensitively to protect the identities of individuals and to avoid the abuse of data, for example, for discrimination or exploitation. Data alone may not be useful to everyone, and training and capacity building may be necessary to enable individuals to discern key insights, as well as translation and dissemination activities to transform data into accessible information. For example, in Mumbai, India, a framework for organizing open-source urban data and training city planners and managers in its use to inform decisions helped to integrate informal settlements into formal urban management and planning processes (Chakraborty et al. 2015). Data collection and information production should not only address the knowledge gap in the design and implementation of policies but also be part of monitoring and evaluation, enabling adaptive and dynamic management that is flexible to changing environmental and social conditions. The availability of novel data sources and processing algorithms, together with innovative forms of governance, shows promise for gathering and using data to support evidence-based transformative climate change adaptation.

## 5 Address the Drivers of Complex Risk

Even more than the formal components of a city, informal settlements face complex risks, meaning they are subject to risks that are tightly interlinked and interact to form compounding and cascading risks and risk accumulations (Westman et al. 2022; Wolff and Ramírez-Lovering 2022; Simpson et al. 2021). Risk accumulation is the buildup of multiple risks due to a combination of political and socioeconomic factors (IPCC, n.d.). It implies that the concentration of exposures (e.g., large, highly vulnerable populations) and the impacts of multiple hazards (climate and non-climate) can change the risk associated with other hazards (Bull-Kamanga et al. 2003). For example, the elevated risk of flooding in Accra, Ghana, has accumulated because of rural–urban migration, unplanned development of low-lying areas, and a lack of drainage infrastructure, which is exacerbated by the marginalization of informal settlements by city authorities (Amoako 2016; Amoako and Frimpong Boamah 2015). Risk compounding occurs when multiple risks interact, simultaneously or sequentially, leading to greater overall impacts (Simpson et al. 2021). For instance, some informal settlements contained some of the highest densities of COVID-19 cases, in part due to shared housing arrangements (Sahasranaman and Jensen 2021). Risk cascading refers to the effect of a hazard triggering another or multiple other hazards in a chain reaction (Wolff and Ramírez-Lovering 2022). For example, in Makassar, Indonesia, flooding disrupts vital services like electricity and water supply, resulting in reduced healthcare provision and a rise in cases of waterborne and infectious disease (Wolff and Ramírez-Lovering 2022). In Medellin, Colombia, informal settlements were shown to be more susceptible to cascading risks than formal settlements, in part due to insufficient and non-resilient critical infrastructure (Purwar et al. 2024).

The complexity of risk in informal settlements means that residents experience significant risk from recurrent and multiple low-magnitude hazards, such as small-scale flooding, that might otherwise (i.e., considered in isolation) not cause major problems. Many such events are expected to become more frequent under climate change (Bull-Kamanga et al. 2003; Nordgren et al. 2016). While all residents of a city may be affected by large-scale events, residents of informal settlements are significantly more likely to be affected by small-scale events because of complex risks, representing another example of the disproportionate impacts of climate change and another pathway through which it can further widen profound inequalities (Satterthwaite and Bartlett 2017). Climate change adaptation represents an opportunity to address complex risk and multiple drivers of risk simultaneously when deployed within the wider context of urban development by exploiting synergies between policy sectors (Pedercini et al. 2019). Decision-makers must identify and address the processes of complex risk that increase the exposure and vulnerability of residents to climate-related hazards. For example, informal settlements are disproportionately exposed to other hazards, including fires, building collapse, disease, and crime, which contribute to their high vulnerability to climate-related hazards (Dodman et al. 2019).

Addressing these non-climate-related hazards could lead to a substantive reduction in multiple and complex risks. For instance, In Kibera, Kenya, instead of focusing on reducing the exposure of residents to specific hazards, multi-sectoral slum upgrading projects that addressed the root causes of vulnerability were the most likely to have long-term success (Mitra et al. 2017). In Bangladesh, climate resilience schemes address the drivers of vulnerability using micro-credit schemes, cooperative interventions, and small-scale entrepreneurial activity (Kisinger and Matsui 2021). Flood evacuation routes in Santo Domingo, Dominican Republic, also give residents safer access to schools (Pelling and Garschagen 2019). However, because the existence of these vulnerabilities and non-climate-related hazards is deeply intertwined with the socioeconomic and political circumstances that led to the formation of the informal settlement to begin with, efforts to address them often face resistance.

Adaptation is often deployed in response to a disaster, after which there can be considerable momentum to restore the status quo, even where this means a return to high levels of risk, and exclusively reduce risk to the most recent hazard (Mochizuki and Chang 2017). However, disasters may represent extraordinary circumstances which offer an opportunity to generate transformative, systemic, and positive social changes, for example toward the central paradigms of both the Sendai Framework for Disaster Risk Reduction (“build back better”) and the Sustainable Development Goals (“leaving no-one behind”) (Ajulo et al. 2020; Patel et al. 2012). Post-disaster circumstances may represent unique periods where political resistance to development in informal settlements is reduced, for example through the guise of transformative climate change adaptation, and where funding for such programs becomes available, for example via internation aid, recovery, and adaptation programs (e.g., the loss and damage fund) (UNFCCC, n.d.). Through pragmatic evidence-based recovery, adaptation programs could address multiple drivers of complex risk, simultaneously reducing risks to climate- and non-climate-related hazards. In practice, a systems approach is needed to identify and address drivers of risk and to inform transformative adaptation measures (Bai et al. 2016). Without this, the prevalent and persistent development pathways that typically characterize cities may remain invisible (Bai et al. 2016). Systems-based approaches can help reveal the complexity of risk in informal settlements (Haimes 2009). Such complexity complicates the development of deterministic forecasts (i.e., a reliable single solution) of how informal settlements will develop or respond to adaptation interventions (Susskind and Kim 2022). Consequently, this must be reflected in the way that adaptation measures are designed and funded, with scenario planning more appropriate than master planning in the context of uncertain climate impacts (Susskind and Kim 2022).

## 6 The Four Principles in Practice

**Figure 3:**
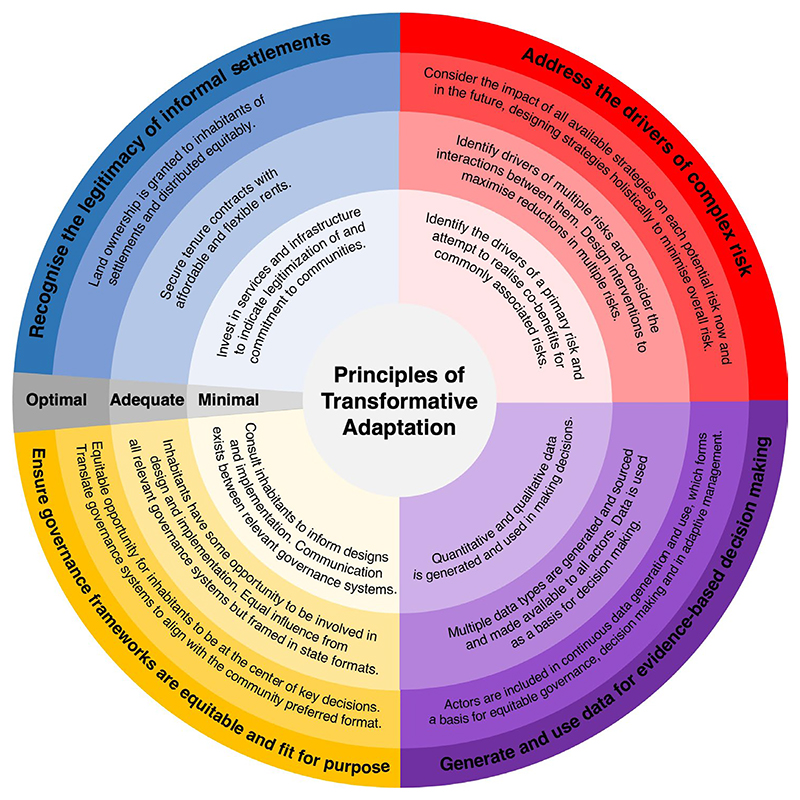
Schematic representation of degrees of achievement of the four principles. Minimal refers to the minimal degree to which a precondition must be achieved to enable transformative adaptation.

Climate change adaptation programs can be complex, multi-faceted, and limited by multiple practical, socioeconomic, and political constraints. As such, while the optimal achievement of each principle remains aspirational, most realistic scenarios will require compromise. We argue that there is a minimal requirement (Figure 3) of each principle that must be met to enable transformative adaptation. We tested this hypothesis on the case studies we identified. Each case study was evaluated to assess the extent to which each of the four principles has been achieved. A scoring system based on the rubric in Figure 3 (where 0 = minimal not achieved, 1 = minimal, 2 = adequate, 3 = optimal) was used to evaluate the case studies. The effects on risk and equity were evaluated, and the overall result of adaptation (regressive, coping, incremental, reformist, or transformative) was determined using the outcomes reported in the studies (Figure 2, Table S1). Practitioners should ensure that at least the minimal requirement is met for every principle and should aim for optimal achievement to maximize the opportunity for transformative adaptation.

Climate adaptation strategies have typically been understood by the actions, policies, and interventions that are used to implement them. For example, common coping strategies include providing emergency relief items following a hazard (Di Baldassarre et al. 2019), and common incremental strategies include building levees and storm drains to reduce flood exposure (Liu et al. 2018). These strategies are favored in practice because they are often arranged in response to a disaster when motivation is highest, they focus on addressing individual challenges, so it is easier to predict, explain, and measure their impact (Few et al. 2017), and they often suit the priorities of the powerful (Kates et al. 2012). However, growing climate-induced pressures such as more severe and less predictable hazards mean these strategies are increasingly ineffective, especially when considered through the lens of inequality because hazards are distributed unequally (Reckien et al. 2023).

Transformative adaptation should not be defined by the policies or interventions themselves but rather by their interactions with socioenvironmental systems. Actions that are transformative in one context can be regressive in another. This makes it difficult to define transformative adaptation by an action or to classify actions as transformative or not, which complicates its application in practice (Few et al. 2017). Instead, transformative adaptation should be understood as a process by which climate change adaptation actions are designed, implemented, monitored, and maintained, and integrated more widely into wider systemic change. In this sense, it is not comparable to other strategies of climate change adaptation (coping, incremental, or reformist) and positioning it as a choice between one or the other is unhelpful.

## 7 Conclusion

Despite international motivation, climate change adaptation is not usually a priority of development programs in informal settlements. Instead, programs more commonly seek to address daily risks to the health and wellbeing of residents, such as shortages of water, food, and medicines, which understandably take precedence over seemingly distant and less frequent climate risks. However, as the frequency and magnitude of climate hazards continue to increase, the impacts on residents of informal settlements will become more severe. Failure to integrate climate change adaptation measures into development programs will increasingly preclude their effectiveness at sustainable poverty reduction. Integrating climate change adaptation into the development of informal settlements is crucial for reducing future risks. Transformative adaptation, which simultaneously advances climate resilience and the sustainable development goals, offers a key pathway to implementing equitable climate change adaptation in these communities.

Transformative adaptation is complex but already achievable through effective collaborations of communities with policy-makers and practitioners who are experienced in sustainable development and climate change adaptation. Such collaborations already contain the skills, knowledge, and experience to co-design transformative approaches, and the actions and policies used to implement them will be familiar. However, we argue for a new emphasis on transformative adaptation as an ongoing process through which climate change adaptation measures are designed, implemented, monitored, and maintained, and integrated into broader systemic change. This implies a considered and long-term approach to governance that commits to building equitable relationships between citizens and informal and formal institutions and adopts systems that are appropriate for the context. Efforts should directly address challenging issues of tenure security that underpin vulnerabilities and discourage progress toward their alleviation. It must address complexity and heterogeneity by maximizing engagement and taking a systems-based approach. A catalyst for these changes can be emerging opportunities for evidence generation and sharing, which serve to unite communities and authorities toward a common goal, push evidence-based and equitable decision-making, and foster flexible and adaptive management in the face of an uncertain future.

The four principles outlined here serve as a baseline for transformative adaptation in informal settlements. Tools such as this and others (e.g., the NAM framework (Reckien et al. 2023)) are vital to accelerate the deployment of transformative approaches. Academics must work closely with communities, practitioners, and policy makers to discern generalizable and context-independent lessons from adaptation attempts in informal settings, and to produce guidance, frameworks, and monitoring networks to enable a scaling-up of transformative approaches.

Climate change adaptation is only effective when it is equitable. On a societal level, adaptation in informal settlements represents the low-hanging fruit of risk reduction, and the context in which transformative adaptation could have the greatest impact. Viewed through this lens, informal settlements become an opportunity to accelerate the rate of adaptation to build a climate-resilient society.

## Supplementary Material

Supporting Information

## Data Availability

Data sharing are not applicable to this article as no new data were created or analyzed in this study.
