# Supplementary material for: Four Principles of Transformative Adaptation to Climate Change-Exacerbated Hazards in Informal Settlements
Source: Wiley Interdiscip Rev Clim Change. Author manuscript; Available in PMC 2025 Jul 20. (PMC7617910; doi:10.1002/wcc.70008)
Supplement: Supporting Information [file EMS206335-supplement-Supporting_Information.docx]

**Four principles of transformative adaptation to climate change in informal settlements**

### Supplementary Information

*Ben C. Howard^1^, Simon Moulds^2^, Sam Agyei-Mensah^3^, Khadiza Tul Kobra Nahin^4^, Zahidul Quayyum^4^, Brian E. Robinson^5^, Wouter Buytaert^1^*

1. *Dept. of Civil and Environmental Engineering, Imperial College London, UK*
2. *School of GeoSciences, University of Edinburgh, UK*
3. *Dept. of Geography and Resource Development, University of Ghana, Ghana*
4. *James P Grant School of Public Health, BRAC University, Bangladesh*
5. *Dept. of Geography, McGill University, Canada*

Examples of climate change adaptation attempts in informal settlements were explored in scientific and grey literature. The aim was not to conduct a systematic review, but instead to identify examples that were of particular relevance to this study. We searched for examples of adaptation to flooding (fluvial, pluvial and coastal), drought, water pollution, heatwaves, and landslides in informal settlements. The search revealed the paucity of examples of adaptation attempts in informal settlements, let alone those which aim for transformative adaptation, and we found no examples of adaptation to heatwaves or drought in informal settlements. Flooding has by far received the most attention, where several examples of both transformative and non-transformative adaptation in informal settlements are available.

Each case study was evaluated to assess to what extent each of the four principles have been achieved. A scoring system based on the rubric in figure 3 (where 0 = minimal not achieved, 1 = minimal, 2 = adequate, 3 = optimal) was used to evaluate the studies. The effects on risk and equity were evaluated and the overall result of adaptation (regressive, coping, incremental or transformative) were determined using the outcomes reported in the studies. The results of this exploration are reported in Table S1.

*Table S1. Published case studies of transformative adaptation attempts in informal settlement, scored using the method described above. The results of the scoring are presented in Figure 2.*

**
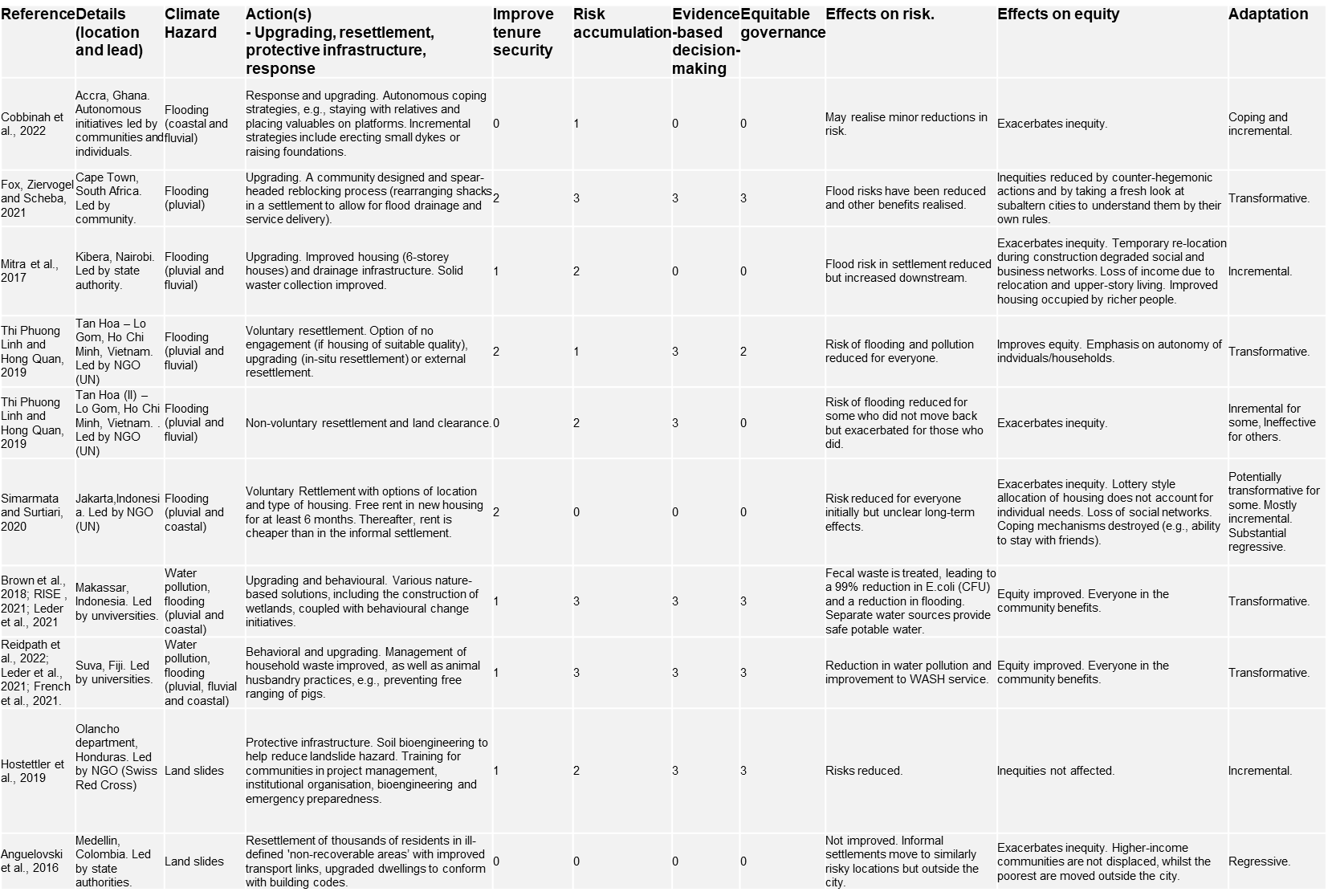
**

**References**

1. Cobbinah, P. B., Asibey, M. O., Boakye, A. A. & Addaney, M. The myth of urban poor climate adaptation idiosyncrasy. Environ. Sci. Policy 128, 336–346 (2022).
2. Fox, A., Ziervogel, G. & Scheba, S. Strengthening community-based adaptation for urban transformation: managing flood risk in informal settlements in Cape Town. *Local Environ.* **0**, 1–15 (2021).
3. Mitra, S. *et al.* Developing risk or resilience? Effects of slum upgrading on the social contract and social cohesion in Kibera, Nairobi. *Environ. Urban.* **29**, 103–122 (2017).
4. Linh, H. T. P. & Quan, N. H. Transformative Adaptation and Social Justice in Ho Chi Minh City, Viet Nam. (2020).
5. Simarmata, H. & Surtiari, G. Adaptation to Climate Change: Decision Making and Opportunities for Transformation in Jakarta, Indonesia. (2020).
6. Brown, R. *et al.* Improving human and environmental health in urban informal settlements: the Revitalising Informal Settlements and their Environments (RISE) programme. *Lancet Planet. Health* **2**, S29 (2018).
7. Leder, K. *et al.* Study design, rationale and methods of the Revitalising Informal Settlements and their Environments (RISE) study: a cluster randomised controlled trial to evaluate environmental and human health impacts of a water-sensitive intervention in informal settlements in Indonesia and Fiji. *BMJ Open* **11**, e042850 (2021).
8. Revitalising Informal Settelements and their environments (RISE) Annual Report 2021. https://www.rise-program.org/__data/assets/pdf_file/0011/2779166/RISE_Annual-Report-2021.pdf (2021).
9. Reidpath, D. D. *et al.* Implementing “from here to there”: A case study of conceptual and practical challenges in implementation science. *Soc. Sci. Med.* **301**, 114959 (2022).
10. French, M. A. *et al.* A planetary health model for reducing exposure to faecal contamination in urban informal settlements: Baseline findings from Makassar, Indonesia. *Environ. Int.* **155**, 106679 (2021).
11. Hostettler, S., Jöhr, A., Montes, C. & D’Acunzi, A. Community-based landslide risk reduction: a review of a Red Cross soil bioengineering for resilience program in Honduras. *Landslides* **16**, 1779–1791 (2019).
12. Anguelovski, I. *et al.* Equity Impacts of Urban Land Use Planning for Climate Adaptation: Critical Perspectives from the Global North and South. *J. Plan. Educ. Res.* **36**, 333–348 (2016).
